# Pilot study to determine differences in breath odour between cigarette and e-cigarette consumers

**DOI:** 10.1038/s41598-022-06047-4

**Published:** 2022-02-09

**Authors:** Annette Dalrymple, Steven Coburn, Marianne Brandt, George Hardie, James Murphy

**Affiliations:** 1 British American Tobacco, R&D, Southampton, Hampshire SO15 8TL UK; 2 Proderm GmbH, Kiebitzweg, 22869 Hamburg, Germany

**Keywords:** Risk factors, Toxicology

## Abstract

Cigarette smoke is known to influence breath odour, but the effect of e-cigarettes is unknown. In this pilot study, we aimed to determine differences in breath odour between cigarette smokers (CSs) and e-cigarette consumers (ECs) in 33 healthy subjects: 11 CSs, 11 ECs and 11 non-smokers (NSs). Breath was sampled at baseline and following product use (CSs and ECs) or a waiting period (NSs) by eight trained odour judges using a six-point smoke intensity scale and a nine-point hedonic scale. We observed a statistically significant difference between CSs and ECs. Smoke intensity values were significantly higher in CSs than ECs and NSs, which were comparable both at baseline and after product use. In addition, hedonic values for CSs were significantly lower than both NSs and ECs after product use. These acute results indicate that cigarette and e-cigarette use results in significantly different breath. ECs breath has a reduced smoke odour and more pleasant aroma than CSs, and is comparable to NSs. This suggests there may be cosmetic benefits for CSs who quit smoking or switch to exclusive use of ECs. Further studies are required to understand the long-term effects of e-cigarette use on breath odour.

## Introduction

The use of e-cigarettes, whose aerosols have reduced levels of toxicants compared to cigarettes, continues to increase globally^1,2^. When CSs switch to exclusive use of e-cigarettes, the level of biomarkers of tobacco exposure such as carbon monoxide, benzene and acrolein are significantly reduce to levels comparable with ex-smokers and NSs^3–5^. In the oral cavity, cigarette smoking can result in gingivitis, periodontitis, tooth loss, epithelial malignancy, tooth staining and breath malodour^6–10^. However, no studies have evaluated the long-term effects on the oral cavity when CSs switch to the exclusive use of e-cigarettes.

The burning of tobacco in a cigarette releases over 7000 chemicals, including a number of known toxicants and volatile gasses^11^. Inhalation of cigarette smoke is thought to be associated with a lingering smoky breath odour^9,12^. Oral malodour may also be attributed to gases produced by oral bacteria. Indeed, differences in oral bacterial populations have been linked to smoking, as has increased breath malodour in smokers^9,12^. E-liquids do not usually contain tobacco, thus their heating within e-cigarettes produces aerosols with significantly fewer compounds than those produced by cigarettes^1,2^. Inhalation of e-cigarette aerosols may therefore result in breath odour that differs from the breath of smokers.

Breath odour can be assessed in the laboratory analytically, however, the use of organoleptic or hedonic assessment by trained judges is thought to be the gold standard. Organoleptic assessments are routinely used to evaluate the effectiveness of malodour reducing mouthwashes or toothpastes^13–16^. In such studies, odour is usually assessed by blinded judges using a six-point intensity scale from 0 to 5, before and after product use^17,18^. In this pilot study, we modify a method used for the assessment of oral care products to assess the breath of ECs and CSs to test the hypothesis that ECs have reduced breath odour. Thirty-three healthy CSs, ECs or NSs were recruited (11 per group) and their breath was assessed by eight trained odour judges using a six-point smoke intensity scale and nine-point hedonic scale. Breath odour following product use (CSs and ECs) or an equivalent period of time (NSs) was compared to baseline values.

## Materials and methods

### Products

All products used in this study are detailed in Table 1 and available for sale in Germany. The cigarettes are a commercial product manufactured by British American Tobacco (BAT) and available for sale in Germany. To enable blind testing, the brand was not printed on the cigarette, the number N491 was printed for product identification. ePen 3 is also manufactured by BAT and is a closed modular rechargeable EC. The e-liquid cartridge used was Blended Tobacco containing 18 mg/ml nicotine. Cigarettes, ePen 3 devices and e-liquid cartridges were stored at room temperature prior to use. ePen 3 devices were charged daily before use.

**Table 1:** Products assessed.

Group	Product used for breath assessment	BAT product code	Source	Consumable/product details
Cigarette	Commercial cigarette blend	N491	BAT, UK	7.2 mg/stick tar 9.2 mg/stick carbon monoxide 0.62 mg/stick nicotine
e-Cigarette	Vype ePen 3	PEN3.0BT18	BAT, UK	Vype ePen Blended Tobacco 18 mg/ml nicotine

### Study design

**Figure 1:**
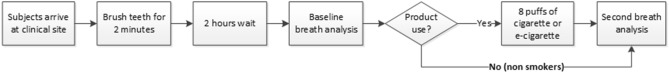
Overview of breath assessment method.

The study was reviewed and approved by the Institutional Review Board of proderm GmbH. Written informed consent was obtained from all individual subjects prior to their participation in the study and before undergoing any study procedures, including screening assessments. The study was conducted according to the main principles of the International Council for Harmonisation (ICH) Guideline for Good Clinical Practice at a single centre in Schenefeld, Hamburg. Two breath evaluations were made per subject, at baseline and following product use. An overview of the study design is detailed in Fig. 1.

### Selection of study participants

Potential subjects attended the study site and were informed about the study by a dentist, who performed an oral and dental examination, recorded their medical history and concomitant therapies, and determined eligibility according to the study’s inclusion/exclusion criteria (Supplementary Table 1). The dental examination confirmed that eligible subjects did not have any oral conditions that would contribute to breath odour and that all had low baseline malodour. Thirty-three subjects were recruited into three groups as detailed in Table 2. Exhaled CO levels were measured using a Bedfont piCOTM Smokerlyzer^®^ to ensure that subjects passed the study inclusion criteria (Supplementary Table 1).

**Table 2:** Subjects recruited for breath assessment.

Group	Subjects per group	Recruitment inclusion criteria
Cigarette smoker	11	Uses manufactured filter cigarettes, excluding menthol
		Minimum of 10 cigarettes per day
		Smoked for at least 3 consecutive years prior to screening
		Exhaled CO-level > 7 ppm at screening
e-Cigarette users	11	Consumes a minimum of 160 puffs per day
		EC user for more than 6 months
		Uses only e-cigarettes
		Exhaled CO-level ≤ 6 ppm at screening
Non-smoker	11	Have never smoked (< 100 cigarettes/oral tobacco products in their life and none within 1 year prior to screening)
		Willing to continue not to smoke or use any form of tobacco for the duration of the study
		Exhaled CO level ≤ 6 ppm

Forty-eight hours prior to assessment day, all subjects were required to refrain from alcohol, coffee, spicy food, garlic and onions. Twelve hours before assessment day, all subjects were obliged to refrain from using lozenges, mouthwashes or chewing gums. On the day of assessment, subjects were asked not to use body lotions, shampoos, hair spray, perfume or lipstick. Two hours prior to arrival for assessment, subjects were permitted to eat breakfast and brush their teeth according to their normal routine. In addition, CSs were permitted to smoke one cigarette and ECs to consume eight puffs of their e-cigarette. On arrival at the site (2 h before baseline assessment), subjects were supplied with a toothbrush and Colgate Komplett 8 toothpaste (Colgate Palmolive, Hamburg, Germany), and supervised by a technician while brushing their teeth for 2 min. No food was permitted during the assessment period; however, subjects could sip water.

### Selection of odour judges

Eight experienced and trained odour judges were selected who matched the inclusion and exclusion criteria (Supplementary Table 2). Odour judges were trained on cigarette and e-cigarette odours using the scales of the study prior the study initiation, following the standard approach for sensory testing, such as explanation of scales, group discussion on samples, effectivity testing of training on further samples. The number of odour judges was in accordance with the International Organization for Standardization ISO 13,299 2016^19^. Written informed consent was obtained from all judges prior to participation in the study and before the initiation of any study procedures, including screening assessments. Prior to assessment, all were required to refrain from alcohol for 48 h and from using body lotions, shampoos, hair spray, perfume or lipstick for 12 h. For 2 h prior to the assessment, all were obliged to avoid tea, coffee, juices, menthol confectionary or chewing gum; they could only drink water during the assessment. Exhaled CO levels of judges were measured using a Bedfont piCOTM Smokerlyzer^®^ and were required to be ≤ 6 ppm.

### Organoleptic assessment

Organoleptic assessment^13–16^ was performed in a dedicated setting, adjacent to the room of the subjects to enabling blind assessment. The assessment of CSs, ECs and NSs breath by odour judges’ was randomised throughout the assessment day. All of the eight odour judges assessed each of the 33 subjects’ breath with a 1-min gap between each judge. The order of odour judges’ assessment remained constant for all subjects, starting with judge 1 and ended with judge 8. By this procedure, the time between product use and odour assessment was comparable for each odour judge and each subject throughout the study. For the assessment of breath, subjects exhaled through a hole in a non-transparent separating wall. Before each odour assessment, subjects kept their mouth closed for 30 s and then exhaled slowly through the hole. On the opposite side of the separation wall, the odour judge smelled the exhaled breath and assigned a value, independent from other judges, using a 6-point smoke intensity scale (Table 3a) and 9-point hedonic scale (Table 3b). Values were inputted into a supplied android tablet using SecuTrial^®^ version 5.4.3.9 software.

**Table 3:** Organoleptic scoring scales for smoke intensity and pleasantness.

	Value
**a: Breath smoke intensity scale**	
Smoke odour could not be detected	0
Questionable smoke odour, barely detectable	1
Slight smoke odour, exceeded the threshold of smoke odour recognition	2
Smoke odour was definitely detected	3
Strong smoke odour	4
Very strong smoke odour	5
**b: Breath pleasantness (Hedonic scale)**	
Extremely unpleasant	− 4
Very unpleasant	− 3
Moderately unpleasant	− 2
Slightly unpleasant	− 1
Neither pleasant nor unpleasant	0
Slightly pleasant	1
Moderately pleasant	2
Very pleasant	3
Extremely pleasant	4

### Breath assessment

Subjects’ baseline organoleptic values were assessed 2 h ± 15 min after teeth brushing. Directly after baseline breath assessment and under the supervision of a technician, CSs smoked 8 puffs of the supplied cigarette and ECs consumed 8 puffs of the supplied e-cigarette. CSs and ECs returned to the study room 5 ± 2 min after product use and organoleptic assessments repeated as described above. NSs did not use any products but had a waiting period of 10 ± 5 min before their second organoleptic assessment.

### Safety precautions

Clinical assessment occurred in January 2020, prior to the COVID-19 pandemic. All procedures were performed according the study protocol and proderm GmbH standard operating procedures. Subjects’ oral and general health, medical history, concomitant therapies, and eligibility according to the study’s inclusion/exclusion criteria were assessed by the study dentist. During breath assessment, clinical staff wore appropriate PPE (gloves and lab coat). Any laboratory consumables or study products that came into contact with a subject’s oral cavity were disposed of according to proderm GmbH standard operating procedures. CO monitors were cleaned according to proderm GmbH standard operating procedures after every use.

### Statistical data analysis

N, mean, standard deviation, median, minimum, maximum and 95% confidence limits were calculated for smoke intensity and hedonic scales. CS, EC and NS groups were independently compared at baseline and after product use using a one-sided t-test for independent samples and a significance level of 0.025 (alpha). The following hypotheses were tested: H0: µA ≥ µB and H1: µA < µB (smoke intensity) and H0: µA ≤ µB and H1: µA > µB (hedonic scale); where µA denotes the mean difference to baseline for treatment A and µB denotes the mean difference to baseline for treatment B. A significance level of 0.05 (alpha) was chosen for this secondary analysis. Comparisons of treatments (CS vs. EC, EC vs. NS) were performed on differences to baseline values for each parameter by a separate t-test for independent samples. Comparisons of assessment times were performed on mean scores for each treatment and parameter by separate paired t-tests. Computation of the statistical data was carried out using commercially available statistics software (SAS for Windows).

## Results

Thirty-three subjects aged 42.4 ± 10.4 years (15 male (45%) and 18 female (55%)) completed the study. No adverse events were documented.

**Figure 2:**
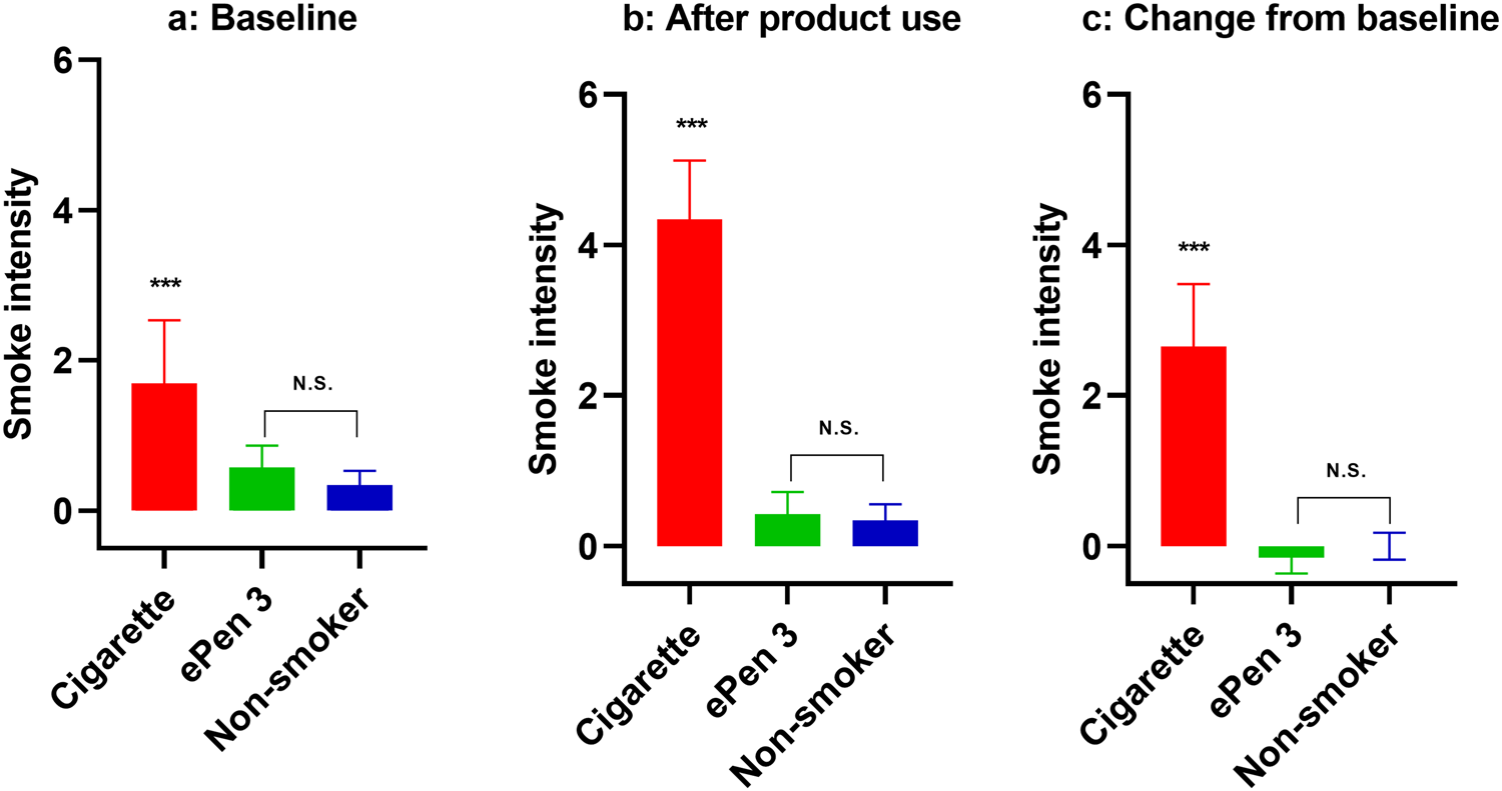
Differences in smoke intensity scoring before and after product use. Values are mean and SD of CSs, ECs and NSs (n = 11 each group) at baseline (**a**), after product use (cigarette and e-cigarette groups only) (**b**) and differences from baseline (**c**). ***p < 0.0001.

In terms of breath smoke intensity at baseline, the CS group had a higher smoke intensity value than either ECs or NSs (p < 0.0001 for both). The breath of ECs and NSs was comparable at baseline. Furthermore, CSs had significantly higher breath smoke intensity after product use than ECs and NSs (p < 0.0001), with no significant difference between ECs and NSs. Each group’s breath intensity after product use was compared to baseline values and significant differences noted in the case of CSs (p < 0.0001) and ECs (and p = 0.041). Both baseline and test values were comparable for NSs. Smoke intensity values at baseline and following product use or an equivalent period of time are detailed in Table 4 and Fig. 2.

**Table 4:** Breath smoke intensity values before and after product use. Mean and SD values at baseline and following cigarette or ePen 3 e-cigarette use. NS values were collected at baseline and 10 ± 5 min later.

Timepoint	Subject group	Baseline mean values (SD)	Differences from baseline mean values (SD)	After product use		
				Compared to e-cigarette	Compared to cigarette	Compared to baseline
Baseline	Cigarette	1.69 (0.84)	–	–	–	–
	e-Cigarette	0.58 (0.29)	–	–	–	–
	Non-smoker	0.34 (0.19)	–	–	–	–
After product use	Cigarette	4.34 (0.78)	2.65 (0.83)	< 0.0001	–	< 0.0001
	e-Cigarette	0.43 (0.29)	− 0.15 (0.21)	–	–	0.0401
	Non-smoker	0.34 (0.22)	0.00 (0.18)	0.0876	< 0.0001	1.0000

**Figure 3:**
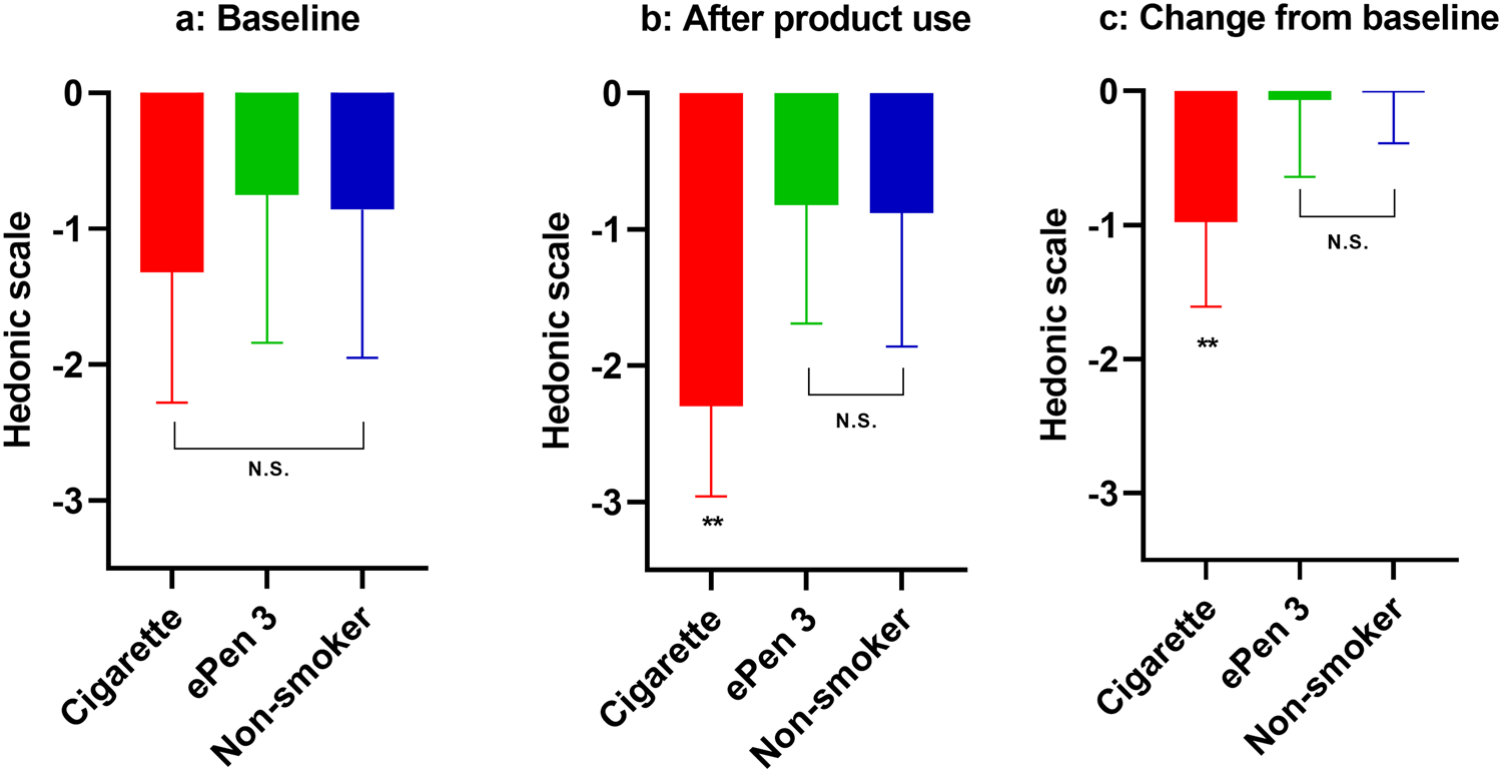
Differences in hedonic scoring before and after product use. Values are mean and SD of CSs, ECs and NSs (n = 11 each group) at baseline (**a**), after product use (cigarette and e-cigarette groups only) (**b**) and differences from baseline (**c**). ** p ≤ 0.001.

Hedonic scale values for CS, EC and NS groups were comparable at baseline. After product use, CSs had significantly lower values than ECs (p = 0.001) and NSs (p = 0.0003). There was no significant difference between the breath of NSs and ECs after product use. When comparing hedonic scale values after product use to baseline values, a significant difference was observed for CSs (p = 0.0004), but not for ECs or NSs. Hedonic scale values at baseline and following product use or an equivalent period of time are detailed in Table 5 and Fig. 3.

**Table 5:** Breath hedonic scale values before and after product use. Mean and SD values at baseline and following cigarette or ePen 3 e-cigarette use. NS values were collected at baseline and 10 ± 5 min later.

Timepoint	Subject group	Baseline mean values (SD)	Differences from baseline mean values (SD)	After product use		
				Compared to e-cigarette	Compared to cigarette	Compared to baseline
Baseline	Cigarette	− 1.32 (0.96)	–	–	–	–
	e-Cigarette	− 0.75 (1.09)	–	–	–	–
	Non-smoker	− 0.86 (1.09)	–	–	–	–
After product use	Cigarette	− 2.30 (0.66)	− 0.98 (0.63)	0.0010	–	0.0004
	e-Cigarette	− 0.82 (0.87)	− 0.07 (0.57)	–	–	0.7004
	Non-smoker	− 0.88 (0.98)	− 0.01 (0.38)	0.7858	0.0003	0.9223

## Discussion

Because inhalation of cigarette smoke is associated with breath odour^9,12^, we devised this pilot study to determine whether differences in breath odour could be measured between CSs, ECs and NSs. Breath odour was assessed before and after product use by trained odour judges using smoke intensity and hedonic scales. NSs formed the control group and did not use a product. Smoke intensity was higher in the breath of CSs compared to either ECs or NSs, both at baseline and following product use. In terms of pleasantness, as measured by the hedonic scale, the breath of CSs was more unpleasant than NSs or ECs after product use. We expected to observe differences in breath pleasantness at baseline, because CSs are known to have odorous breath^9,12^, but all groups were comparable before product use. There was variability in the hedonic data for all groups, possibly due to the low subject numbers assessed, thus a difference might be observed at baseline if higher subject numbers were assessed.

The breath of ECs and NSs was comparable both before and after product use, suggesting that e-cigarette use does not affect the breath of consumers by inducing a noticeable malodour. These data support published studies^9,12^ and confirm a stronger smoke odour and increased breath unpleasantness in CSs. The aerosol formed from tobacco burning in a cigarette is thought to cause the lingering smoke malodour associated with smokers’ breath^9,12^. E-cigarettes produce aerosols with significantly fewer compounds than a cigarette^1,2^. The data in this study support the hypothesis that inhalation of e-cigarette aerosols results in breath odour that differs to CSs.

Other analytical methods have been used to assess compounds in the breath of CSs and ECs. Solid-phase microextraction–gas chromatography/mass spectrometry demonstrated significant differences between CSs, ECs and NSs^20^. In addition, thermal desorption coupled to gas chromatography/mass spectrometry was used to assess room air and breath following cigarette or e-cigarette use, revealing that EC exhaled breath had similarities to room air and significantly differed from CSs^21^. Both these studies support our finding that the breath of CSs differs from ECs.

As e-cigarettes are a relatively new product, few studies have looked at the long-term effects on the oral cavity. A clinical study that assessed subjects for 120 days after switching from smoking cigarettes to e-cigarettes observed reductions in bacterial plaque on teeth surfaces^22^, which suggest that switching to e-cigarettes results in a change in the oral bacteria population than found in smokers. Such a change in bacterial population could in turn change volatile sulphur compound (VSC) levels, which are known to be associated with smokers’ breath malodour^9,12^. Improved smell and taste, indicating changes in the oral cavity and olfactory neurons, have also been linked to smokers switching to e-cigarette use^22^. Furthermore, an in vitro study that assessed enamel staining up to 86 days after product exposure found that staining levels were significantly less with e-cigarette than cigarette exposure, and similar to the study control^23^.

The current study was performed prior to the 2020 COVID-19 pandemic. The organoleptic methods employed here may increase the risk of virus transmission and are therefore unsuitable until the end of current pandemic. In the absence of organoleptic assessment, VSC levels could be measured in breath using an OralChroma™^24^, Halimeter™^25^ or Breath Alert™^26^. VSCs are produced in the mouth by gram-negative anaerobic oral bacteria^27^ and are higher in the breath of smokers^25^ due to their unique population of oral bacteria^12^. This is exacerbated by the VSCs that are present in cigarette smoke^25^. Prior to this pilot study, we performed a preliminary assessment of the levels of VSCs in CS, EC and NS breath using an OralChroma™. The data obtained were extremely variable, possibly due to subjects using their own product, and no conclusions could be made (unpublished data). The current study was designed to incorporate organoleptic assessment, which is the gold standard for breath assessment^28^, and subjects were supplied with a specific product. If restrictions continue to limit the use of organoleptic assessment, a future study could be performed using the OralChroma™ and specific products supplied to CSs or ECs with the aim of reducing data variability.

There are a number of limitations in the current pilot study. For example, subject number was low per group, 8 puffs per product was tested and subjects used their own cigarette or e-cigarette brand prior to assessment, which could result in data variability. This study also assessed subjects breath at one time point, therefore the long-term effect of e-cigarette use to breath odour is still unknown. Future studies could be planned with increased subject numbers, specific products supplied for a number of days prior to breath assessment and breath assessed over a number of weeks/months. In addition, because smoking is known to affect the gums and cause gingivitis and periodontitis, additional endpoints that assess gum health could enable a better understanding of the long-term impact of switching from cigarettes to exclusive use of e-cigarettes. However, compliance would need to be monitored to ensure the switching population did not combine e-cigarette use with cigarette smoking^29^.

## Conclusions

Our study confirms that cigarette and e-cigarette use results in breath with significant differences in aroma. Compared to CSs, the breath of ECs had reduced smoke odour, was more pleasant and was also comparable to the breath of NSs. These short-term results suggest there may be cosmetic benefits for CSs who quit smoking or switch to exclusive use of e-cigarette. Further studies are required to understand the effects of e-cigarette use on the oral cavity and if switching exclusively to of e-cigarette results in a long-term improvement to breath odour.

## Supplementary Information

Supplementary Tables.

## References

[1] Margham J (2016). Chemical composition of aerosol from an e-cigarette: A quantitative comparison with cigarette smoke. Chem. Res. Toxicol..

[2] Tayyarah R, Long GA (2014). Comparison of select analytes in aerosol from e-cigarettes with smoke from conventional cigarettes and with ambient air. Regul. Toxicol. Pharmacol..

[3] Cravo AS (2016). A randomised, parallel group study to evaluate the safety profile of an electronic vapour product over 12 weeks. Regul. Toxicol. Pharmacol..

[4] Makena P, Liu G, Chen P, Yates CR, Prasad GL (2019). Urinary leukotriene E4 and 2,3-dinor thromboxane B2 are biomarkers of potential harm in short-term tobacco switching studies. Cancer Epidemiol. Biomark. Prev..

[5] Shahab L (2017). Nicotine, carcinogen, and toxin exposure in long-term e-cigarette and nicotine replacement therapy Users: A cross-sectional study. Ann. Intern. Med..

[6] Alandia-Roman CC, Cruvinel DR, Sousa AB, Pires-de-Souza FC, Panzeri H (2013). Effect of cigarette smoke on color stability and surface roughness of dental composites. J. Dent..

[7] Bergström J (2004). Tobacco smoking and chronic destructive periodontal disease. Odontology.

[8] Dalrymple A (2018). Assessment of enamel discoloration in vitro following exposure to cigarette smoke and emissions from novel vapor and tobacco heating products. Am. J. Dent..

[9] Hanioka T (2019). Smoking and periodontal microorganisms. Jpn. Dent. Sci. Rev..

[10] Stratton, K., Shetty, P., Wallace, R. & Bondurant, S. *Institute of Medicine (US) Committee to Assess the Science Base for Tobacco Harm Reduction. Clearing the Smoke: Assessing the Science Base for Tobacco Harm Reduction*. (National Academies Press US, 2001). 25057541

[11] Perfetti TA, Rodgman A (2013). The Chemical Components of Tobacco and Tobacco Smoke.

[12] Bazemore R, Harrison C, Greenberg M (2006). Identification of components responsible for the odor of cigar smoker's breath. J. Agric. Food Chem..

[13] Hu D (2003). Clinical effectiveness of a triclosan/copolymer/sodium-fluoride dentifrice in controlling oral malodor: A three-week clinical trial. Compend. Contin. Educ. Dent..

[14] Schmidt NF, Tarbet WJ (1978). The effect of oral rinses on organoleptic mouth odor ratings and levels of volatile sulfur compounds. Oral Surg. Oral Med. Oral Pathol..

[15] Sharma NC (1999). The clinical effectiveness of a dentifrice containing triclosan and a copolymer for controlling breath odor measured organoleptically twelve hours after toothbrushing. J. Clin. Dent..

[16] Sharma NC (2002). The clinical efficacy of Colgate Total Plus Whitening Toothpaste containing a special grade of silica and Colgate Total Toothpaste for controlling breath odor twelve hours after toothbrushing: A single-use clinical study. J. Clin. Dent..

[17] Li Y (2019). A randomized parallel study to assess the effect of three tongue cleaning modalities on oral malodor. J. Clin. Dent..

[18] Wigger-Alberti W, Gysen K, Axmann EM, Wilhelm KP (2010). Efficacy of a new mouthrinse formulation on the reduction of oral malodour in vivo. A randomized, double-blind, placebo-controlled, 3 week clinical study. J. Breath Res..

[19] International Organization for Standardization: ISO 13299:2016 Sensory analysis—Methodology—General guidance for establishing a sensory profile. https://www.iso.org/standard/58042.html (2016). Accessed 14 May 2019.

[20] Papaefstathiou E, Stylianou M, Andreou C, Agapiou A (2020). Breath analysis of smokers, non-smokers, and e-cigarette users. J. Chromatogr. B Anal. Technol. Biomed. Life Sci..

[21] Marco E, Grimalt JO (2015). A rapid method for the chromatographic analysis of volatile organic compounds in exhaled breath of tobacco cigarette and electronic cigarette smokers. J. Chromatogr. A.

[22] Tatullo M, Gentile S, Paduano F, Santacroce L, Marrelli M (2016). Crosstalk between oral and general health status in e-smokers. Medicine.

[23] Dalrymple A (2021). Enamel staining with e-cigarettes, tobacco heating products and modern oral nicotine products compared with cigarettes and snus: An in vitro study. Am. J. Dent..

[24] Szabó A (2015). Volatile sulphur compound measurement with OralChroma(TM): A methodological improvement. J. Breath Res..

[25] Jiun IL (2015). Association between oral hygiene status and halitosis among smokers and nonsmokers. Oral Health Prev. Dent..

[26] Khurana C, Tandon S, Chinmaya BR (2018). A crossover clinical trial to assess the effectiveness of different oral hygiene regimens on the reduction of morning bad breath in healthy young adults. Indian J. Dent. Res..

[27] Jamali Z, Alipour M, Ebrahimi S, Aghazadeh M (2019). Effect of Halita mouthwash on oral halitosis treatment: A randomized triple-blind clinical trial. J. Dent. Res. Dent. Clin. Dent. Prospects.

[28] Greenman J (2004). Study on the organoleptic intensity scale for measuring oral malodor. J. Dent. Res..

[29] Camacho, O. M. *et al.* Use of the acrylonitrile haemoglobin adduct *N*-(2-cyanoethyl)valine as a biomarker of compliance in smokers switching to tobacco heating products. *Preprints* 2021080085 (2021).

