# Supplementary material for: Pilot study to determine differences in breath odour between cigarette and e-cigarette consumers
Source: Sci Rep. 2022 Feb 9;12:2204. doi: 10.1038/s41598-022-06047-4 (PMC8828822; doi:10.1038/s41598-022-06047-4)
Supplement: Supplementary file 1 — Supplementary Tables. [file 41598_2022_6047_MOESM1_ESM.docx]

# Supplementary Table1: Inclusion and exclusion criteria for subjects

| **Inclusion criteria** | **Group** |
| --- | --- |
| Female or male, 21 to 60 years of age |  |
| Written Informed Consent to participate in the study | All |
| Willingness to actively participate in the study and to come to the scheduled visits | All |
| Understand and comply with the requirements of the study | All |
| Average oral hygiene (tooth brushing at least twice a day) | All |
| Good general health as judged by the Investigator | All |
| Regular cigarette smoker of manufactured filter cigarettes, excluding menthol cigarettes.  Have smoked for 3 consecutive years prior to screening and smoke at least 10 cigarettes per day  CO-level >7 ppm at screening | Cigarette smokers |
| Regular e-cigarette consumer and willing to use a test e-cigarette  Consumes a minimum of 160 puffs per day for more than 6 months prior to screening  Uses only e-cigarettes with CO-level ≤6 ppm at Screening | E-cigarette consumers |
| Have never smoked or < 100 cigarettes in their life and none within 1 year prior to screening  Willing to continue not to smoke or use any form of tobacco for the duration of the study  CO-level ≤6 ppm at Screening | Non-smokers |
| **Exclusion criteria** |  |
| Female subjects: Pregnancy or lactation | All |
| Drug or alcohol addiction | All |
| AIDS, HIV or infectious hepatitis if known to the subjects | All |
| Participation in similar cosmetic and/or pharmaceutical studies | All |
| Pathological changes of the oral mucosa or gingival (e.g. allergic reactions, ulceration candidiasis) | All |
| Active caries | All |
| Current periodontitis or non-physiological tooth mobility (mild gingival inflammation accepted) | All |
| Use of any medication, food supplementation, homeopathic therapy which could cause malodour | All |
| Xerostomia caused by medication (e.g. anticholinergics, antidepressants, antipsychotics) | All |
| Ongoing dental treatment or any other treatment of the oral cavity (e.g .oral appliances) | All |
| Oral prophylaxis treatment within the last week or during the study | All |
| Current chronic heartburn or other chronic stomach problems | All |
| Antibiotics within 14 days prior to screening or during the study | All |
| Pain medication 3 days before or during the study | All |
| "roll your own" tobacco cigarettes, chewing tobacco or snuff/snus, dual-users of a cigarette and e-cigarette, or dual-users of a cigarette and tobacco heating product | All |
| Employees and immediate relatives of the tobacco industry or the clinical site | All |
| History of or acute tuberculosis | All |

# Supplementary Table 2: Inclusion and exclusion criteria for Odour Judges

| **Inclusion criteria** | **Group** |
| --- | --- |
| Female or male 18 to 65 years of age |  |
| Written Informed Consent to participate in the study | All |
| Willingness to actively participate in the study and to come to the scheduled visits | All |
| Understand and comply with the requirements of the study | All |
| Have never smoked or < 100 cigarettes in their life and none within 1 year prior to screening  Willing to continue not to smoke or use any form of tobacco for the duration of the study  CO-level ≤6 ppm at Screening | All |
| Willingness not to drink alcohol 48 hours prior to assessments day | All |
| Willingness not to use products that might affect the senses (e.g. body lotions, shampoos, perfume and lipstick) 12 hours prior to and at the assessments |  |
| Willingness to abstain from consuming spicy food, garlic, onion or alcohol within the last 48 hours prior to odour assessments | All |
| **Exclusion criteria** | All |
| Female subjects: Pregnancy or lactation | All |
| Drug or alcohol addiction | All |
| AIDS, HIV or infectious hepatitis if known to the subjects | All |
| Conditions which exclude a participation or might influence the test reaction/evaluation for example upper respiratory tract infection | All |
| Consuming spicy food, garlic, onion or alcohol within the last 2 days prior to odour assessments | All |
